# Supplementary material for: Modeling interconnected minerals markets with multicommodity supply curves: examining the copper-cobalt-nickel system
Source: Nat Commun. 2025 Aug 7;16:7302. doi: 10.1038/s41467-025-62570-8 (PMC12332016; doi:10.1038/s41467-025-62570-8)
Supplement: Supplementary file 1 — Supplementary Information [file 41467_2025_62570_MOESM1_ESM.pdf]

Supplementary Information for:

Modeling interconnected minerals markets with multicommodity supply curves: examining the copper-cobalt-nickel system

John Ryter*[1], Karan Bhuwalka[2], Richard Roth[3], Elsa Olivetti[4], Laura Buarque-Andrade[5], Max Frenzel[5], Ensieh Shojaeddini[1,6], Elisa Alonso[1], Nedal Nassar[1]

1: U.S. Geological Survey, National Minerals Information Center, Reston, VA, USA; **2**: Stanford University, Precourt Institute for Energy, Stanford, CA, USA; **3**: Massachusetts Institute of Technology, Materials Systems Laboratory, Cambridge, MA, USA; **4**: Massachusetts Institute of Technology, Department of Materials Science and Engineering, Cambridge, MA, USA; **5**: Helmholtz-Zentrum Dresden-Rossendorf, Dresden, Germany ; **6**: Akima System Engineering, Contractor to the U.S. Geological Survey, Reston, VA, USA

**To whom correspondence may be addressed: Email address: jryter@usgs.gov; Mailing address: 12201 Sunrise Valley Dr., Mail Stop 998, Reston, VA, USA 20192*

Description:

This document contains additional detail for the methodology, including the theoretical framework for a three-commodity system, the one-dimensional method as used in this work, and detailed descriptions of the data used, including modifications and gap filling. The document also contains detail surrounding the historical tuning process and the method for selecting the lowest-error scenario. Finally, the document contains a table comparing the methods used in this work with the prior best practice. The data repository accessible at https://doi.org/10.5066/P13KPFRL contains the raw data generated in this study and the underlying data for each figure and table in the main text. Data for figures and tables in the Supplementary Information may be available upon request.

Table of Contents

Table of Contents 2
Supplementary Information 3
1 Methods 3
1.1 Theoretical framework for a three-commodity system 3
1.2 Applying the one-directional approach to a multi-commodity system 6
1.3 Production, total demand, and secondary demand data sources 6
1.4 Mine data initialization 8
1.5 Filling production gaps 10
1.6 Imputing missing cost data 12
1.7 Homogenizing mine-level data sources 13
1.8 Price data and mine cost adjustments 15
1.9 Mine-level operation 15
1.10 Secondary supply 16
1.11 Production, consumption, and inventory evolution 17
1.12 Calculating equilibrium prices 19
1.13 Historical tuning and selection process 21
2 Results 22
2.1 Performance comparison with other methods 22
References 23

Supplementary Information

1 Methods

1.1 Theoretical framework for a three-commodity system

The process of finding the supply-demand-price equilibrium becomes more complicated in a three-commodity system. In our example, nickel is now included alongside copper and cobalt, again using the 50 cobalt-producing mines for which cost data was available from S&P Capital IQ Pro,[1] representing 69.3% of 2023 cobalt production. With three commodities capable of influencing free cash flow, a cash flow plane as shown in the two-commodity case exists for every possible value of the third-commodity price, in this case nickel. The sets of cutoff or marginal commodity price combinations, where free cash flow=0, are no longer described using a line as in the two-commodity case, but would now be represented using a plane. Similarly, to visualize supply quantities for each commodity as a function of three commodity prices, an additional dimension is required beyond the two-commodity case. Supply curves exist for every possible value of the third-commodity price, as shown in Supplementary Figure 1.

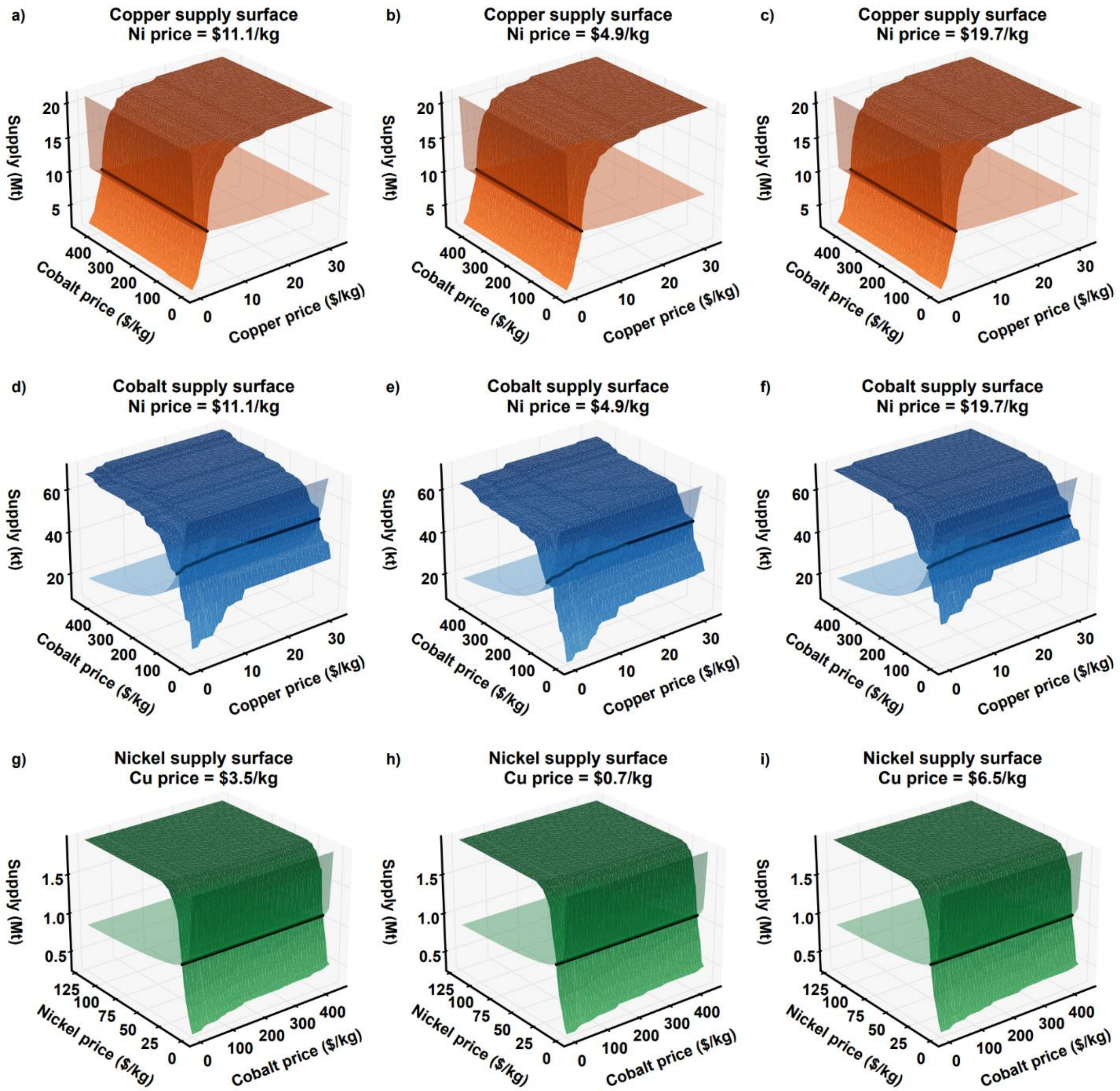

Supplementary Figure 1. Supply and demand surfaces for a three-commodity system, showing copper (top row), cobalt (middle row), and nickel (bottom row). Each column uses a different nickel price except for the nickel row, where each column is a different copper price. a) copper at moderate nickel price; b) copper at low nickel price; c) copper at high nickel price; d) cobalt at moderate nickel price; e) cobalt at low nickel price; f) cobalt at high nickel price; g) nickel at moderate copper price; h) nickel at low copper price; i) nickel at high copper price. Mt = million metric tons; kt = thousand metric tons; $/kg = United States dollars per kilogram.

It is evident from Supplementary Figure 1 that nickel prices have a decided impact both on the height and shape of the cobalt supply surface (panels d, e, and f), but no effect on the copper supply surface (panels a, b, c). Copper price has a negligible effect on the nickel supply surface, only evident in the shape of the bottom edge of the supply surface (Supplementary Figure 1 g, h, i). As in the two-commodity case, demand surfaces are treated independently for each commodity, but equivalent surfaces now exist for all values of the third commodity price. With differing supply surfaces for each

nickel price, each cobalt supply surface will thus have a separate curve describing the intersection of the supply and demand surfaces and therefore supply-demand equilibrium.

As in the two-commodity case, the intersection of each pair of supply-demand curves for fixed third-commodity price produces a new curve that varies along all three axes (Supplementary Figure 2a). Again, these curves represent supply-demand equilibria as functions of the two prices shown, and with a fixed price for the third commodity. Creating supply-demand intersection curves for all values of the third commodity's price creates a surface comprised of intersection curves, also representing equilibrium.

Plotting these supply-demand surface intersection curves across all third-commodity prices (e.g. the nickel or copper prices corresponding to columns in the figure above), the supply-demand equilibrium lines for each commodity produce surfaces that are functions of all three prices, rather than the curves seen in the two-commodity case. The three commodity-specific surfaces have a single, unique intersection point, as illustrated in Supplementary Figure 2a. Finally, this equilibrium point may be plotted on each supply surface, where it is evident that for each commodity, this point lies on the supply-demand surface intercept curve, given the equilibrium third-commodity price (Supplementary Figure 2b-d)

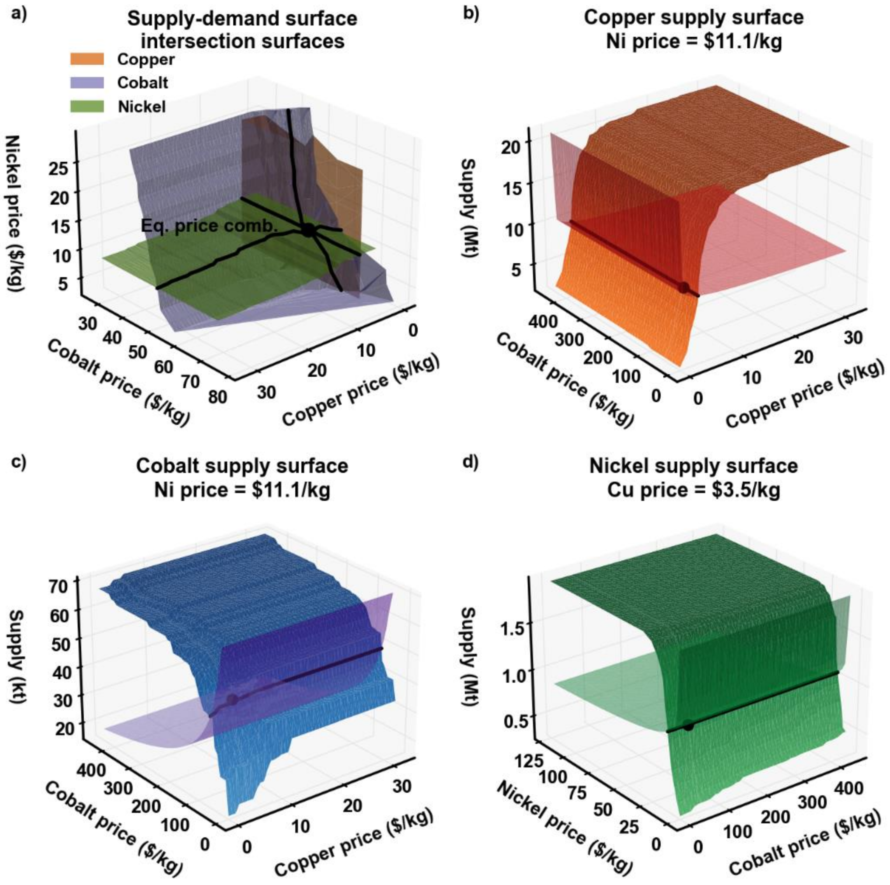

Supplementary Figure 2. a) The surfaces representing the intersection between the supply and demand surfaces across all third-commodity prices, showing the black intersection curve between each pair of surfaces and the point where all three curves and surfaces intersect. b), c), and d) The supply and demand surfaces for copper, cobalt, and nickel at the equilibrium third-commodity price (\$12.3/kg for Ni, \$6.5/kg for Cu), showing the curve of supply-demand surface intersection and the supply-demand-price equilibrium point in black. Mt = million metric tons; kt = thousand metric tons; \$/kg = United States dollars per kilogram; Eq. price comb. = equilibrium price combination.

While visualization becomes impossible as dimensionality (the number of commodities) increases, the method developed in this work for the calculation of these intersection points is sufficiently general to enable any number of commodities. Consequently, the calculation time for obtaining equilibrium price and supply scales roughly as A^n, where A is some constant and n is the number of commodities in the system.

1.2 Applying the one-directional approach to a multi-commodity system

The one-directional approach, implemented as in Nguyen et al.,[2] uses coproduct-basis (revenue-based) cost accounting to construct supply curves for each commodity independently. Within each year, the revenue fraction of each commodity at each mine is calculated using historical or most recent available prices. The mine is assigned to its respective supply curve based on the largest revenue fraction among cobalt, copper, and nickel. In Nguyen et al., copper and nickel mines that do not produce cobalt were assumed to produce only copper or nickel respectively. In the dataset used in this work, substantial copper production exists at nickel-primary mines. To maintain a consistent mine dataset across the two methods explored in this work, copper production at nickel mines was assumed to behave as a byproduct, and the nickel supply curve was solved first. The breakeven price was calculated for each mine m at time t and commodity c according to Supplementary Equation 1.

$$\text{breakeven price}_{m,t,c} = \frac{\text{ore treated}_{m,t}\ \text{cost}_{m,t}\ \text{revenue fraction}_{m,t,c}}{\text{production}_{m,t,c}} \qquad 1$$

Mines were arranged in order of smallest breakeven price, and the cumulative production associated with each of these breakeven prices produces the supply curve. The equilibrium price was then found using the intersection of the supply and demand curves as described in the previous section. For copper, this process was repeated. However, for nickel-primary mines, copper breakeven prices were assigned based on nickel profitability. If the nickel-primary mine had already been found profitable based on the nickel equilibrium price, the mine also produced copper; otherwise, the mine did not produce nickel or copper. The process was repeated for cobalt, with the mining decision made by the primary commodity as described above.

1.3 Production, total demand, and secondary demand data sources

Data for total demand, mine production, and secondary demand was calculated by taking the median value for each year across all data sources listed in Table 1 of the main text, with resulting values shown in Supplementary Figure 3. Secondary demand information was the least reliably available, and in many cases represents the estimated secondary recovery. Given that reporting of secondary production and consumption is variable, secondary production and consumption were assumed equivalent. For cobalt, the Cobalt Institute reports estimated secondary recovery of cobalt for 2013-2020,[3] Darton reports

refinery secondary feedstocks for 2017-2020,[4] Project Blue reports world feedstocks for 2013-2050,[5] Roskill reports estimated secondary recovery 2013-2019,[6] and Sun et al. report the results of a dynamic material flow analysis (dMFA) for 2000-2015,[7] giving estimated secondary supply (Supplementary Figure 3c). For copper, the International Copper Study Group reports secondary feed into smelter production for 2001-2019,[8–11] while for Klose & Pauliuk we subtract primary supply from total demand to estimate secondary supply, providing data from 2017 to 2050 (Supplementary Figure 3f).[12] For Nickel, Elshkaki et al. use dMFA to estimate secondary supply,[13] the International Nickel Study Group reports nickel scrap input in stainless steel production (assuming other sources of nickel scrap are negligible) for 1995-2020,[14] and Project Blue reports scrap consumption in stainless steel and batteries for 2013-2050,[15] where battery scrap consumption is 2 kt or less prior to 2018 and 20 kt or less prior to 2022 (Supplementary Figure 3i). Projections for scrap supply or demand were available from several sources through 2034 or later, but these are withheld in Supplementary Figure 3 due to the proprietary nature of the data and the more singular availability of such projections. From these projections, mean projected annual demand growth for cobalt ranges from 3.8% to 6.6%, for nickel from 4.7% to 6.2%, and for copper only one growth rate was identified at 1.7% (Supplementary Figure 3a, g, and d, respectively). Mine production data are shown in Supplementary Figure 3 b, e, and h for cobalt, copper, and nickel, respectively.

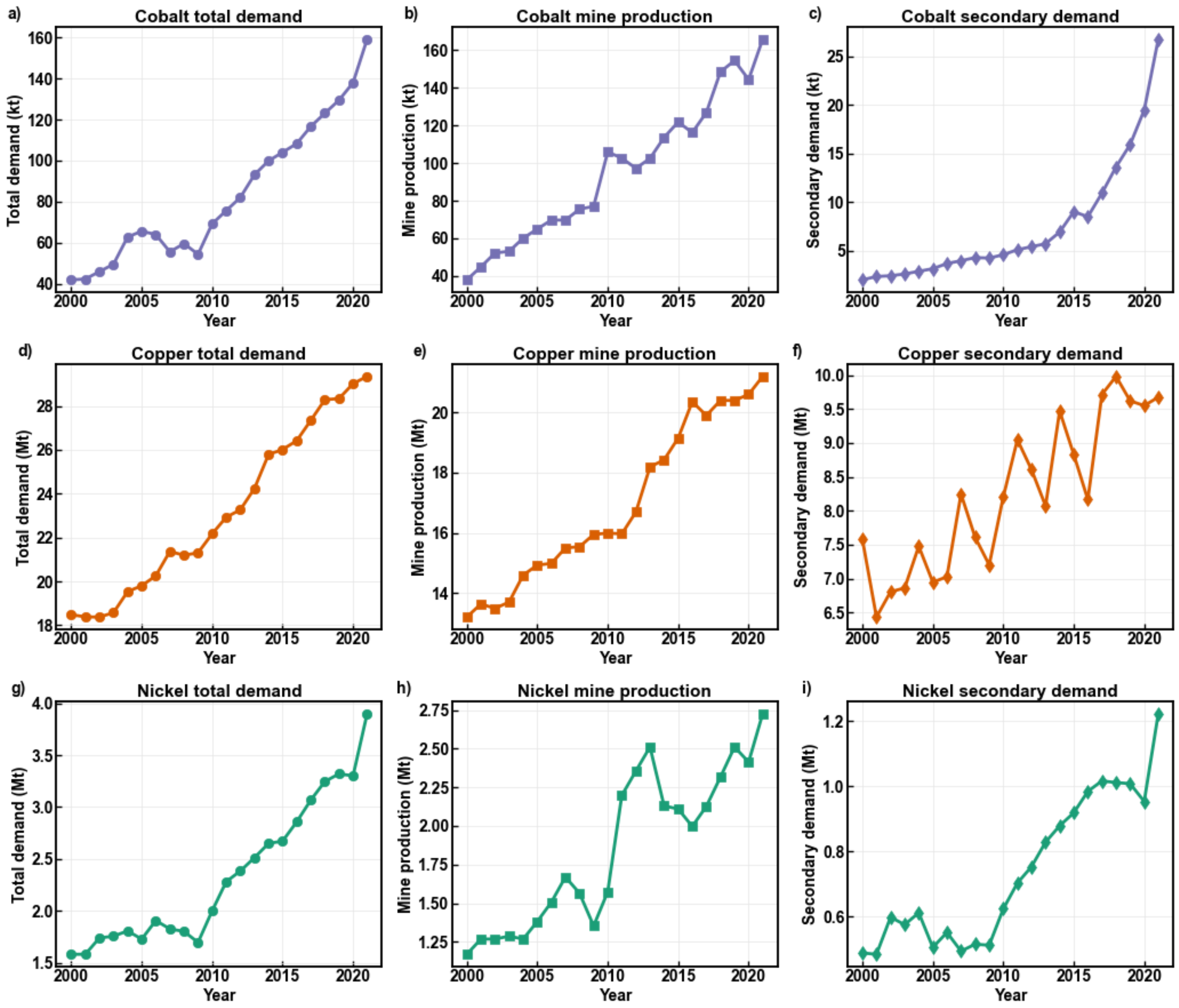

Supplementary Figure 3. Total demand, mine production, and secondary demand, 2001-2021. Top row: cobalt, middle row: nickel, bottom row: copper. Left column: total demand, center column: mine production, right column: secondary demand. a) cobalt total demand; b) cobalt mine production; c) cobalt secondary demand; d) copper total demand; e) copper mine production; f) copper secondary demand; g) nickel total demand; h) nickel mine production; i) nickel secondary demand. Mt = million metric tons; kt = thousand metric tons.

1.4 Mine data initialization

The S&P Capital IQ Pro Metals and Mining Properties Screener contains annual mine-level, commodity-level production, cost, ownership, and location information, both as reported by each mining property and as modeled by S&P Capital IQ Pro.[1] Particularly for production, the inclusion of modeled data can result in double counting, and consequently production data relies on reported data alone. Data were imported for all mines producing copper, nickel, or cobalt in any year, for the years 1991-2040. Collecting forward-looking data enables inclusion of mines S&P Capital IQ Pro forecasts will come online. However, this data set may not cover all mines undergoing feasibility studies or other development, and may underestimate future supply availability, particularly beyond the immediate future. Where production from the assembled mine dataset did not match total production, additional mines were

generated to fill the gap using a resampling with perturbation method described in Supplementary Section 1.5. Within the screener, reported data are categorized as actual, best-of, estimate, and forecast; where data were available for multiple categories, values were prioritized in order of actual, best-of, estimate, forecast.

Supplementary Table 1. Mine characteristic names used by the model with corresponding labels for the reported and modeled data in the S&P Capital IQ Pro Metals and Mining Properties Screener and in the S&P Capital IQ Pro Capital Costs Screener, as well as the basis on which the data were provided and used within the model. Cap. = capital; LOM = life of mine; CU = capacity utilization; $/t = U.S. dollars per metric ton.

**Resulting column name**	**Reporting basis**	**Column name, reported**	**Column name, modeled**	**Column name, cap. costs screener**
Production (kt)	Mine, commodity, year	Commodity Production – tonne (tonnes)	N/A	LOM Yearly Production – tonne/y (tonnes)
Total Cash Cost ($/t ore treated)	Mine, year	Mining & Processing Costs per tonne ($/tonne)	Total Cash Cost ($/t ore treated)	Mining & Processing Costs per mt ($/tonne)
Ore Treated (kt)	Mine, year	Ore Processed Mass (tonnes)	Ore Treated (kilotonnes)	Mill Capacity – tonnes/year (tonnes/yr), multiplied by 80% CU estimate
Reserves (kt)	Mine, year	Reserves: Ore Tonnage (tonnes)	N/A	Reserves: Ore Tonnage (tonnes)
Head Grade (%)	Mine, commodity, year	MillHead Grade – percent (%)	Head Grade (%)	LOM Head Grade - % (%)
Recovery Rate (%)	Mine, commodity, year	Recovery Rate (%)	Recovery Rate (%)	N/A
Priority: Reported (Actual → Best of → Estimate → Forecast) → Modeled → Capital Costs Screener				

Additional data sources were included to capture mines in earlier stages of development such as feasibility studies. A secondary data source was the S&P Capital IQ Pro Capital Costs Screener,[16] which contains feasibility study data including much of the same data as the Metals and Mining Properties screener but on a life-of-mine average basis rather than a yearly basis. These values were used for all years the mine could be operating, starting with the earliest year among the projected completion year, actual completion year, projected start up year, and actual start up year columns. The average annual ore treated was assumed to be 80% of the mill capacity, using an approximate average of mine capacity utilization within the Metals and Mining Properties Screener data. With data reported across many feasibility studies for these mines, the data corresponding to the most advanced study was used, in this order: mine plan, full feasibility, prefeasibility, preliminary economic assessment.

Additional data sources included Gulley,[17] Project Blue,[5,15] and WoodMackenzie.[18] Among these, only the Gulley et al. dataset had sufficient historical data for inclusion as an aid in filling production gaps, going back to 2001, while Project Blue and WoodMackenzie only had data for 2011 and 2013 onward, respectively. As a result, the latter two data sources were used for identifying future mine projects only. In all cases, yearly production data were provided alongside mine name, owner and/or operator name, and country, and were combined to match mines duplicated across sources (Supplementary Section 1.7)

For mines without data coverage for reserves, mines operating at simulation start were initialized with either the earliest reported ore reserves for that property, or the reserves reported at simulation start, if available, summed with any increases in reserves that occur over the life of the mine. New mines were assigned the earliest reported reserves, also adjusted to account for any increases. Reserves are reduced by the quantity of ore treated after each year in which that mine produces. When the ore treated quantity for a given year exceeds the remaining reserves, the ore treated quantity is set to the quantity of remaining reserves and production quantities for each commodity are scaled by the ratio between the quantity of remaining reserves and the initial ore treated quantity. When reserves are exhausted, that mine may no longer operate. This method does not include any method for mine-level reserve expansion; rather, expansions are emulated via the opening of new mines. Reserves, with additions associated with indicated reserve expansions, were used in place of resources because they represent the currently economical resource and are the values used to estimate mine life.

For mines where no total cash cost data were available, mean values were imputed using a K-nearest neighbors approach with the following exogenous variables: mean commodity-level production, mean ore treated, primary mine type (underground, open pit, placer, tailings, etc.), and global region (North America, Africa, etc.), with more details in Supplementary Section 1.6.

1.5 Filling production gaps

Because mine production from compiled data was less than USGS values for the simulation start date, additional mine production was added via resampling from existing mines. There are three steps in this process, each of which introduces a level of randomness. First, mines are sampled such that smaller mines are more likely to be included, given small mines are more likely missing from the data set than larger mines. Second, each numerical value for each mine is randomly multiplied by some value in the uniform distribution $U(0.9,1.1)$ to provide a $\pm$10% perturbation. Finally, a linear programming optimization algorithm selects perturbed mines to fill the production gaps for each year and commodity simultaneously, minimizing the difference with historical production. Mines are selected from the initial resampling described below to be operating at reported levels in each year.

The initial resampling is performed as follows. For each year in the historical simulation period, each mine is randomly assigned a value from a uniform distribution $U(0,1)$, where the random seed may be varied. Each mine’s mean production across all years of production is normalized by the mean mine production across all years for each commodity. Due to the presence of years with very small production gaps to fill for some commodities and very large gaps for others, the normalization was performed separately for mines producing multiple target commodities and those producing only one, with the normalization factor for mines producing only one target commodity multiplied by 2 to give small normalized production and higher likelihood of selection. This normalized production value was then passed through an exponential probability density function (PDF), which returns high values for low production values and lower values for high production values. Each mine is then selected for resampling if its exponential PDF value is greater than the randomly-assigned uniform distribution value for any of the commodities it produces. If the production associated with the subset of mines is less than the production gap for any commodity in any year, the exponential PDF value is multiplied by 1.1 until the production gap is filled. Each numerical value for each mine is then perturbed as described above.

With each mine producing multiple commodities, random selection could not produce total production values reliably close to those from USGS,[19–23] so a linear programming optimization algorithm was implemented to select mines to minimize this production gap. The subset of mines is passed to this algorithm, which minimizes production for each commodity and year with the constraint that subset mine production must exceed the gap for each commodity and year, as described in the Supplementary Equations 2-3.

$$\text{Minimize}\left(\sum_y^Y \sum_c^C \sum_m^M Q_{y,c}\left(\alpha_{m,y} P_{m,y,c} - G_{y,c}\right)\right) \text{ where } \left(\sum_m^M \alpha_{m,y} P_{m,y,c}\right) - G_{y,c} > 0 \;\forall\; y, c \tag{2}$$

$$Q_{y,c} = \frac{\min\limits_{c} G_y}{G_{y,c}} \tag{3}$$

Where $Q_{y,c}$ is a scaling factor to weight each commodity's production gap such that a sum over all commodities weighs all commodities' production gaps at the same scale for each commodity c in the set of commodities C and each year y in the simulation period, $\alpha_{m,y}$ is a Boolean parameter with value either 0 or 1 indicating whether mine m in the subset of mines M is selected for inclusion in year y, $P_{m,y,c}$ is the production of commodity c in year y at mine m, and $G_{y,c}$ is the difference between mine production from the compiled data and the USGS production data for commodity c in year y. The optimization is implemented using a "brute force" method which creates 20,000 random orderings of the subset of mines for each year, iteratively performing a cumulative sum of production and dropping all mines where the cumulative sum exceeds the first value above $G_{y,c}$ for any commodity, iterating until all commodities' gaps are satisfied or no additional mines remain. From the 20,000 selections from the subset of mines generated, the one satisfying the minimization in the Supplementary Equation 2 is selected, giving the value of $\alpha_{m,y} = 1$ for each mine in the selection. After performing this method for all years, these mines are appended to the initial set of compiled data with production only in the years where $\alpha_{m,y} = 1$. These mines' costs are multiplied by 0.7 to limit their potential effect on the upper end of the cost curve and limit the likelihood these mines act to set prices.

Supplementary Figure 4 shows the mean mine-level production and cost histograms for all mines used in the simulation, split by the original mines compiled from the dataset and the supplementary mines generated via resampling with perturbation to fill the gap between original mine production and reported production. Supplementary production distributions are skewed to the left relative to the original mines, as it was assumed that the mines missing from the dataset were small, and the resulting mine cost distributions are skewed to the right relative to the original mines. Supplementary nickel mines are relatively plentiful relative to original mines due to low production coverage in the original dataset.

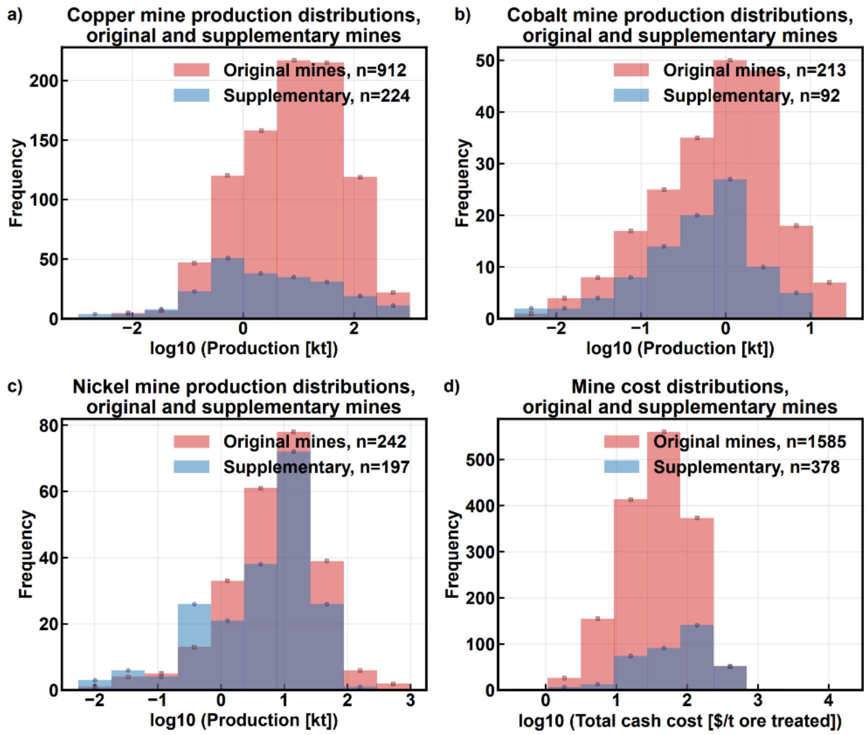

Supplementary Figure 4. Mine production distributions for a) copper, b) cobalt, and c) nickel, as well as d) mine cost distributions for all mines producing copper, cobalt, or nickel. The mean value for each mine is shown to avoid skewing data due to multiple years of operation. Red is for the original set of mines from the compiled data, while blue is for the supplementary mines selected to fill the gap between production from the original set of mines and USGS reported production. The number of data points associated with each histogram are shown in the figure legend as n. kt = thousand metric tons; t = metric tons.

1.6 Imputing missing cost data

Where there is production reported yet no costs, cost data was imputed from other available data where possible. Rather than attempt to impute each year's cost data, imputation was performed using mean ore treated-basis costs for each mine for training, with the goal to impute the logarithm of mean costs. The following parameters were used for the imputation: logged mean commodity-level production, logged mean ore treated, primary mine type (underground, open pit, placer, tailings, etc.), and global region (North America, Africa, etc.). The k-nearest neighbors approach was used with the number of neighbors set to 3 due to its error minimization as shown in Supplementary Figure 5 below. The approach was implemented using the *KNNImputer* class in the *scikit-learn* package in Python, which outperformed the random forest regression approach used in the *scikit-learn IterativeImputer* class.[24]

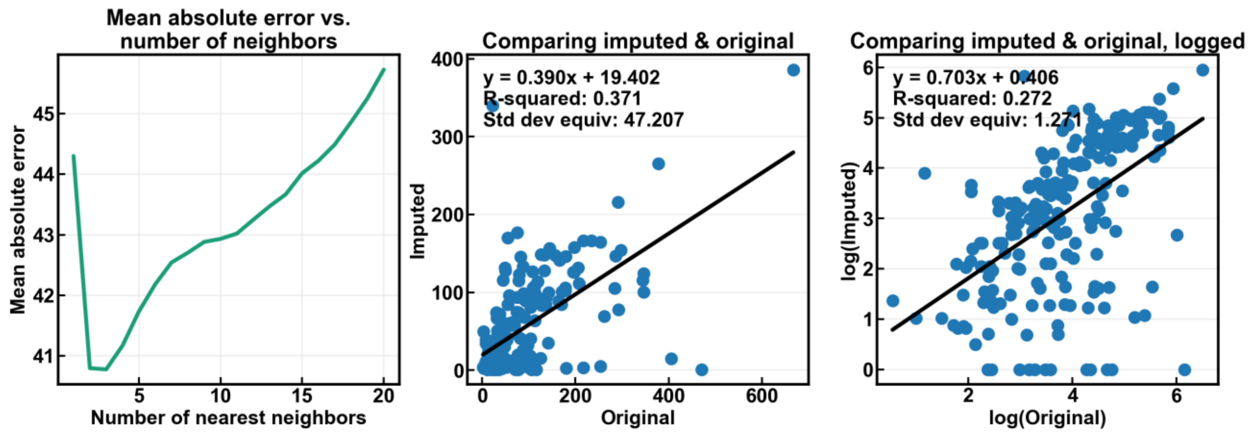

Supplementary Figure 5. The standard deviation equivalent shown in b and c is the square root of the mean squared error for the residuals.

1.7 Homogenizing mine-level data sources

The primary data source for mine production and costs is the S&P Capital IQ Pro Metals and Mining Properties Screener,[1] supplemented by feasibility study data in the S&P Capital IQ Pro Capital Costs Screener,[16] and mine-level data from Gulley,[17] Project Blue,[5,15] and WoodMackenzie.[18] The differing sources of mine data were combined to the extent possible, where WoodMackenzie and Project Blue provide production and forecasts for many cobalt and nickel properties and Gulley et al. data provided production for many cobalt mines, including artisanal production. WoodMackenzie and Gulley et al. data provided cobalt production only, while Project Blue provides both cobalt and nickel production. WoodMackenzie also provided production forms or products, as well as the primary commodity for each mine. Primary commodities for Project Blue were inferred from whether the mine was included in the nickel production data, where properties not on the nickel production set were assumed copper primary. Primary commodities for the Gulley et al. data were provided, but artisanal mining operations were assumed to produce only cobalt.

Because mine names were reported differently for each data source, the matching process was accelerated using the *fuzzywuzzy* package in Python,[25] which uses the Levenshtein Distance to calculate the difference between each property name.[26] For each mine in the Gulley et al., Project Blue, and WoodMackenzie datasets, names were compared against all names in the S&P Capital IQ Pro database, including the “Also Known As” column, and those from the WoodMackenzie and Project Blue data respectively. The most similar mine names within the target mine country that produced the target commodity were selected for comparison, verification, and assignment. Each match was verified manually, and all unmatched mines were checked for alternative names. Finding none, mine properties were compared with any associated with their owners/operators in S&P Capital IQ Pro databases. For mines that were found not to be present in the S&P database following this process, new mine identifiers were given and their production, region, and joint products were appended to the full mine data set wherever possible. For mines identified to be in multiple databases, the median production value across all three databases in each year was selected.

The Gulley et al., WoodMackenzie, and Project Blue data sets did not include ore treated, reserves, or primary commodity production (except Project Blue nickel) data. Ore treated for each mine in each year was calculated by dividing the production for each mine and year by the mean head grade for the corresponding commodity across all mines and years in the S&P Capital IQ Pro data set. Where production for multiple commodities was provided, the largest resulting ore treated value was used. Initial reserves were calculated by generating values for each mine from a normal distribution $N(15,5)$ where any values less than 5 were replaced by the mean to represent the number of years the mine may be open, then multiplying by the maximum ore treated value for each mine over all years. Mean values were preferred over the arbitrarily chosen mean due to the uncertainty in this parameter and the reality that many mines identify new reserves over their lifetimes, an aspect which is not otherwise accounted for in this work. For primary commodity production, if the primary commodity did not match the commodity with production reported, the primary commodity production was estimated by multiplying the reported production for each year by a production ratio between the reported commodity and primary commodity from the S&P Capital IQ Pro data set. This production ratio was the mean production ratio for a randomly selected mine from among all mines with reported commodity production and the same primary commodity. Due to the greater potential for error in these values relative to production, these values were only added to the database if no values were reported in the S&P Capital IQ Pro sources, rather than taking the median-based approach described for production above. This approach avoids dramatic changes in ore treated, reserves, or primary commodity production values, since empty values in the database are filled with earliest available or most recent values as described in the "Mine-level operation" section.

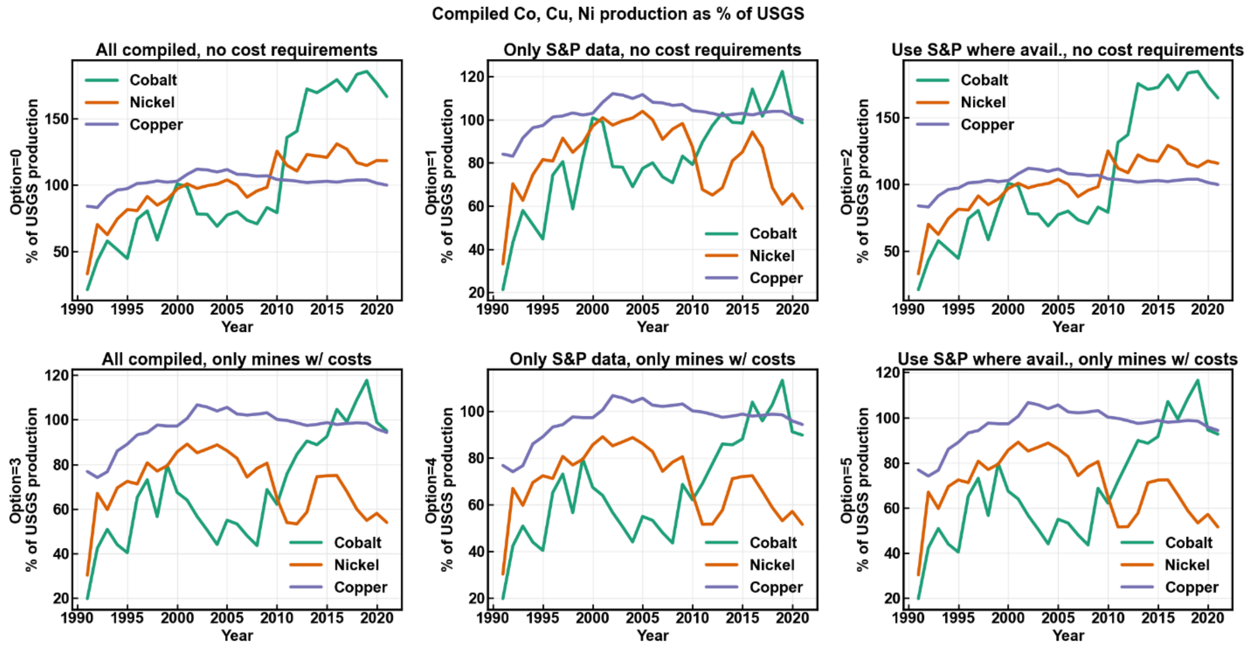

Supplementary Figure 6. Mine production for Co, Ni, and Cu as a percent of the USGS reported production, varying which mines are included (differing by row) and how their production is calculated (differing by column). Leftmost column: using the median production values for individual operations across S&P, Project Blue, and WoodMackenzie; center column: excluding Project Blue and WoodMackenzie data; right column: using S&P data even when Project Blue or WoodMackenzie production values are available. Top row: sum of production for all mines with production reported; bottom row: sum of production for all mines where any total cash cost is reported. USGS = United States Geological Survey; w/ = with.

The top row in Supplementary Figure 6 differs substantially across columns due to differences in data availability from different sources. The bottom row is minimally different across columns because Project Blue and WoodMackenzie do not report mine cost information, and thus all properties that are not reported in S&P are excluded. Nickel production is particularly low in the bottom row and for the top row S&P data-only case because the S&P database does not report ferronickel production under either the nickel label or ferronickel label, despite both being present.

1.8 Price data and mine cost adjustments

To ensure that any possible joint production at all mines was accounted for in free cash flow calculations, price data was obtained from S&P Capital IQ Pro for chromite, cobalt, copper, gold, graphite, lead, lithium, manganese, molybdenum, nickel, palladium, platinum, ruthenium, silver, and zinc. Inflation adjustment was performed using the world average producer price index (PPI) from World Bank,[27] which was reported monthly at the country level. To produce the world average, this monthly PPI was normalized to August 2022, the last month for which US data was available, and the average over all countries was taken. Commodity prices were reported on a daily basis, and were averaged to a monthly basis for compatibility with PPI. For mine costs, the global PPI was averaged to an annual basis and applied to each mine and year.

For all commodities except cobalt, copper, and nickel, prices were assumed equal to historical values after adjusting for inflation, and were fixed at the most recent year's inflation-adjusted value for all future years. Daily price values were converted to monthly mean prices, inflation adjustment was performed at the monthly timescale, and inflation-adjusted monthly values were averaged to give annual prices.

1.9 Mine-level operation

Each mine is assigned a year in which it becomes available for opening based on the earliest of the following three years: the actual opening year as reported in S&P Capital IQ Pro, the projected opening year as reported in S&P Capital IQ Pro, and the earliest year in which production data were available in S&P Capital IQ Pro or other sources. Mines projected to open later than 2021, the end of the historical testing period, have their earliest year available decreased by one to account for uncertainty in opening timing. For all simulation years greater than the resulting available year, the mine is included within the equilibrium price calculation above, entering production if it has a positive free cash flow at the equilibrium price. Mines produce at historical or projected levels, shifted by any misalignment between historical or projected start up year and the simulated start up year. Mines available for opening are assumed to have completed the construction phase of mine development, and are waiting only for sufficiently high prices to generate positive free cash flow in that year. Mines open immediately with no development capital expenditure. These are substantial simplifications that could be addressed in future work. Other methods have calculated the break-even price needed to enable a particular internal rate of return,[28] but the free cash flow approach has been used for simplicity.

The production value is then the production reported in that year if available, the earliest reported production reported if the modeled mine opens earlier than data is available, or the most recent reported production otherwise. Ore treated is considered in the same way, while the mean total cash cost per tonne of ore treated over its lifetime was used for each mine. Operating mines are permitted to operate with negative free cash flow for one year prior to closure, regardless of cash available in previous years. This approach emulates the inertia associated with placing a mine on care and maintenance or shutting down. Once a mine has had negative free cash flow for more than one consecutive year, it is placed on care and maintenance and returns to the set of potential opening mines unless its reserves are exhausted, at which point it closes. No costs are allocated to these actions, and reserve expansion is not modeled.

1.10 Secondary supply

Given this paper seeks to highlight a novel modeling method, a simplistic approach to secondary supply was used. Assuming the secondary supply and secondary demand reported in our dataset represent the maximum scrap availability, the recyclable material available in a given year was assigned costs at different fractions of total scrap supply, using the secondary supply fraction-cost combinations developed in Nguyen et al.[2] The supply curve shapes were assumed to be constant for all years, where the maximum secondary production costs were $76.5/kg, $7.15/kg, and $19.8/kg for cobalt, copper, and nickel, respectively. Within the supply surface method, each cost level-quantity combination for secondary supply was included as a separate supply source, capable of producing its fraction of that year's scrap demand only in that year and at the given cost.

Supplementary Figure 7 shows the total free cash flows for each source of supply for 2001, providing a visualization of the secondary supply available and its profitability relative to primary supply sources. Cash flows for primary supply sources include cash flows associated with all commodities produced at the mine. Cash flows were computed using the modeled 2001 values for price in the 4D approach, being $45/kg, $7.1/kg, and $11.1/kg for cobalt, copper, and nickel, respectively. Treating secondary supply sources as "mines," ore treated values were set equivalent to production values such that the free cash flow in Equation 4 in the main text would treat costs on a production basis as they were reported rather than the ore treated basis used by all primary supply sources. This method mirrors that of Nguyen et al.[2]

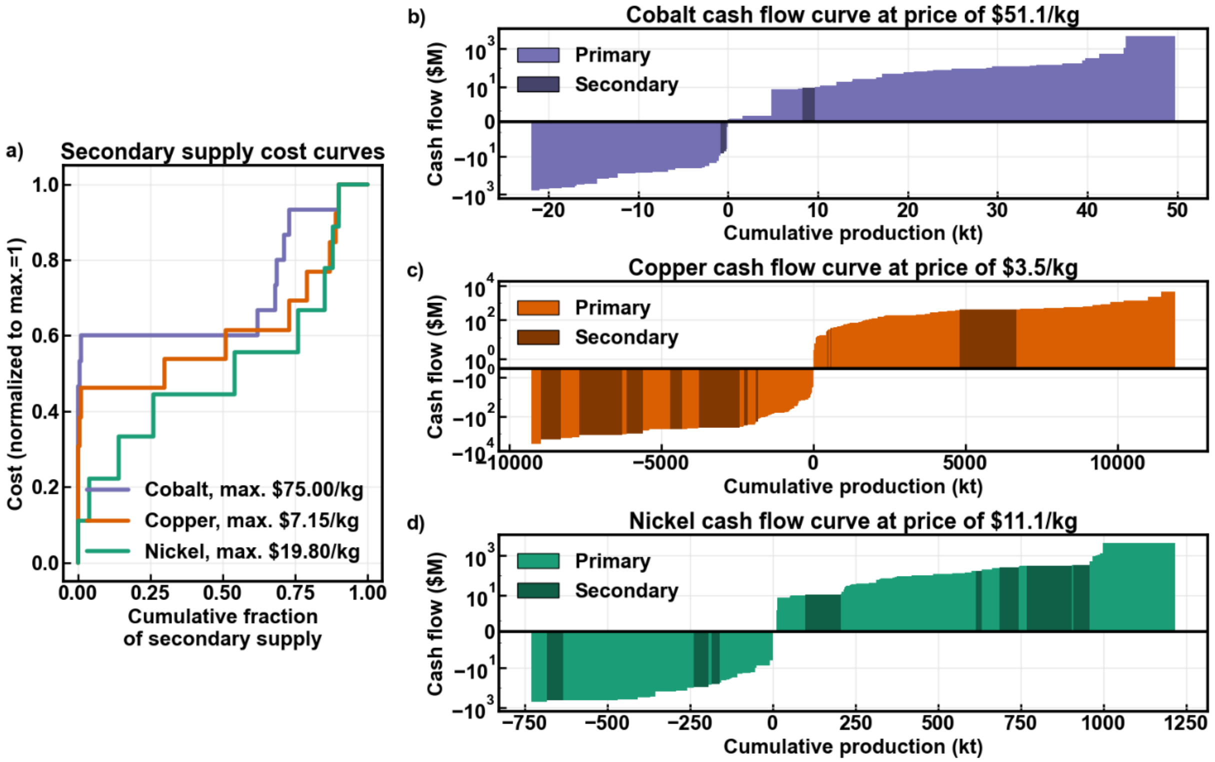

Supplementary Figure 7. a) Cost curves for secondary supply following the approach used by Nguyen et al. [2]. Right: total cash flow curves at fixed 2001 equilibrium prices for b) cobalt ($51.1/kg) (c) copper ($3.5/kg) and d) nickel ($11.1/kg) showing cumulative production for all mines as a function of free cash flow, for 2001. Primary supply includes all mines producing that commodity and uses the combination of these prices corresponding to the commodities produced at each mine; many mines are present on multiple cash flow curves. Negative production values correspond with cash flows below zero, showing the amount of production that does not come online in 2001. Primary supply sources are shown in lighter colors; secondary supply sources are shown in darker colors. Data is transformed using the normalized symmetrical base-10 logarithm as implemented in *matplotlib* [29] . $M = million U.S. dollars; max. = maximum; kg = kilogram; kt = thousand metric tons.

1.11 Production, consumption, and inventory evolution

For the individual supply curves created by holding all other commodities' prices constant at the equilibrium price, there are two possible ways it can intersect with the demand curve. First, the supply and demand curves can intersect in a vertical section of the supply curve, as shown in the top row of the figure below. In this case, production and consumption are treated as equivalent, while the equilibrium price is well above the operating cost of the most expensive supplier, indicating higher profits in that year. In the second case, the demand curve intersects a horizontal part of the supply curve, as shown in the second and third rows of Supplementary Figure 8. Given production is discrete, it cannot exactly match the value of the supply-demand curve intersection. The production value for each commodity is thus equal to $\text{Total Production}_{\text{optimal}}$ corresponding to the value of p from Supplementary Equation 10, price is the corresponding $\text{supply price}_{\text{optimal}}$, and consumption is the value on each demand curve corresponding with $\text{supply price}_{\text{optimal}}$. Imbalances in production and consumption are thus permitted in the model and changes in inventory are simply tracked, with no additional effects. The resulting production and consumption values are shown alongside the supply and demand curves in

Supplementary Figure 8. Because consumption must lie on the demand curve, consumption is determined by the x-axis value of the supply-demand intersection, while price is determined by the y-axis value of this intersection. The method finds smallest difference between the price determining supply and the demand-curve price associated with that supply; as a result, production is determined by the nearest vertical part of the supply curve on the x-axis.

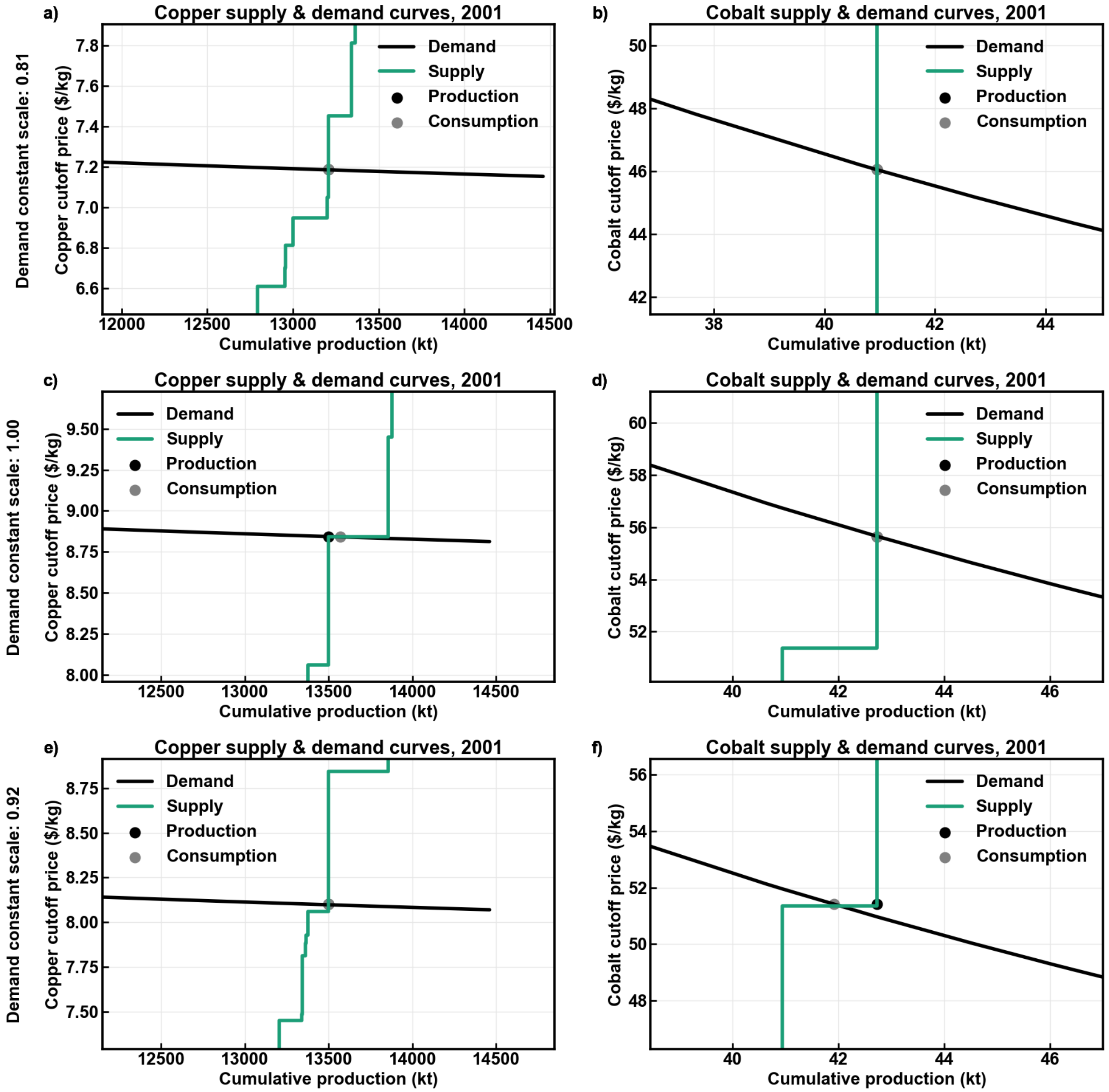

Supplementary Figure 8. Examples of different intersections between the supply and demand curves for copper (left column: a, c, e) and cobalt (right column: b, d, f), where the intersection occurs in the vertical part of the supply curve (top row (a, b)), the left side of a horizontal part of the supply curve (middle row (c, d)), and the right side of a horizontal part of the supply curve (bottom row (e, f)). Production is determined by the nearest vertical part of the supply curve on the x-axis, consumption by the x-axis value of the supply-demand intersection, and price by the y-axis value of this intersection.

The handling of demand curve intersections with horizontal sections of the supply curve leaves two issues unresolved. In some cases, as in Supplementary Figure 8f, this approach places the resulting price-production combination used as an approximation of supply-demand equilibrium above the demand curve. The value of consumption for this price is thus below production, indicating oversupply and thus a decline in price, indicating that the last mine on the supply curve should sustain a loss. However, in the case that consumption exceeds production, as in Supplementary Figure 8c, there is undersupply which should incentivize a higher price than determined by the current method. Both these issues could be solved by additional iterations where the price suggested by the demand curve is used to calculate production after each iteration; however, given the three commodities' interconnected production and prices, this additional iteration could push the system farther from the point nearest equilibrium across all commodities, potentially leading to an unsolvable system or a lack of convergence. This method does not account for stockpiling or changes in capacity utilization, both of which could also address these issues by removing the discrete nature of the existing supply curves.

1.12 Calculating equilibrium prices

While equilibrium prices can be calculated by finding the intersections between each supply and demand surface and then the intersections between the resulting curves and planes as described in Supplementary Section 1.1, a more general and computationally efficient approach is used for model operation, described below. For each mine site available in each year, free cash flow is calculated in line with the multi-commodity free cash flow equation (Equation 4, main text), for all combinations of a set of commodity prices, in this case a set of N=50 prices ranging from zero to 10 times the current or most recent reported price for each commodity. The price set thus consists of 50^n price combinations, where n is the number of commodities considered, e.g., 3 for the copper-cobalt-nickel system. Mines are considered operating for a given price combination if their free cash flow is greater than zero or if their free cash flow was positive in the preceding year, if they operated in the preceding year. As a result, the total production associated with each price combination is the sum of production from all mines that were profitable in the preceding year and the production from all mines where free cash flow is greater than zero for that price combination, as shown in Supplementary Equation 4 for each mineral commodity c.

$$\text{Total Production}_{c,p} = \sum_{m \in \text{mines}} \text{Production}_{m,c} * \max[\delta(\text{free cash flow}_{m,p}), \delta(\text{last free cash flow}_{m,p})] \quad 4$$

$$\text{where } \delta(x) = \begin{cases} 1 \; if \; x > 0 \\ 0 \; else \end{cases} \quad 5$$

Where $p \in (1, 50^n)$ represents the index of the price combination of length n under consideration in $\text{supply price}_{c,p}$, which is the matrix containing all price combinations. For mines that were not producing in the preceding year, $\text{last free cash flow}_{m,p}$ is treated as negative; the $\text{free cash flow}_{m,p}$ for the current year must be positive. Next, asserting that total production must equal demand for supply-demand equilibrium, the price associated with each production value from the demand side is calculated using Supplementary Equation 6.

$$\text{demand price}_{c,p} = \alpha_c \left(\text{Total Production}_{c,p}\right)^{1/\beta_c} \quad 6$$

Where $\text{demand price}_{c,p}$ is the demand-side price combination for commodity c resulting from the demand value equal to $\text{Total Production}_{c,p}$ and price combination p, α_c is the demand shifter for commodity c, and β_c is the demand elasticity. Demand elasticity values were held constant over time and were selected from those reported by Shojaeddini et al. for cobalt and nickel,[30] with copper derived following the same method (manuscript under preparation). The exponent is $1/\beta_c$ because the reported price elasticity of demand describes the demand response to price, the inverse of Equation 1 in the main text. The static price elasticity of demand was used, giving values of -0.45 for cobalt, -0.05 for copper, and -0.09 for nickel.

Because the calculated $\text{Total Production}_{c,p}$ stems from a unique combination of prices ($\text{supply price}_{c,p}$) where this value can then be used to calculate a separate combination of prices ($\text{demand price}_{c,p}$), the equilibrium point is reached when $\text{supply price}_{c,p} = \text{demand price}_{c,p}$ for all commodities. Because the calculations performed here are discreet and obtaining this equality is computationally expensive, the squared difference between $\text{supply price}_{c,p}$ and $\text{demand price}_{c,p}$ is minimized instead (Supplementary Equation 7).

$$\text{SE}_{c,p} = \left(\text{demand price}_{c,p} - \text{supply price}_{c,p}\right)^2 \qquad 7$$

Where $\text{SE}_{c,p}$ is the squared error for each commodity and price combination. Each commodity's squared error is normalized by the largest SE for each commodity to enable a sum across all commodities on the same scale as shown in Supplementary Equations 8-10.

$$\text{SE}_{c,\text{max}} = \max_p(SE_c) \qquad 8$$

$$\text{SNSE}_p = \sum_{c=0}^{n} \frac{\left(\text{demand price}_{c,p} - \text{supply price}_{c,p}\right)^2}{\text{SE}_{c,\text{max}}} \qquad 9$$

$$\text{SNSE}_{\text{optimal}} = \min_p(\text{SNSE}) \qquad 10$$

Where SNSE_p is the sum of normalize squared errors for each price combination, and the value of p corresponding with the minimum SNSE value can be used to retrieve the optimal values for supply price, demand price, and Total Production.

While total production should ideally be equal to demand, the supply curve is not continuous due to the discrete nature of the operating decision for each mine. As a result, the supply-demand intersection occurs where the supply surface is vertical or horizontal, and additional corrections are required (Supplementary Section 1.11). Although production and consumption are tracked independently, this method does not consider metal inventories or their evolution.

To ensure sufficient price and supply resolution, the process is repeated using all possible combinations of a new set of 50 price values for each commodity, with the new values for $supply\ price$ within $\pm$20% of the optimal prices found in the first iteration. This additional iteration, in a two-commodity example, reduced the maximum difference (across commodities) between $\text{supply price}_{\text{optimal}}$ and

$\text{demand price}_{\text{optimal}}$ from 12% to 0.2%, and additional iterations could be added to reduce this error further. In this case, the 41 lowest-error price combinations produce equivalent production values, indicating the method is beyond the threshold for inaccuracy due to a lack of granularity. For a three-commodity example, this additional iteration reduced the maximum difference between $\text{supply price}_{\text{optimal}}$ and $\text{demand price}_{\text{optimal}}$ from 12% to 0.65%, and the lowest-error 22 price combinations produce equivalent production values. The value of $\text{supply price}_{\text{optimal}}$ was used in the simulation because it ensures that only mines with cashflows greater than zero can produce.

1.13 Historical tuning and selection process

The Bayesian optimization method was used to tune the demand curve parameters and reproduce historical production and price as closely as possible. Bayesian optimization works by placing a Gaussian process prior on the objective function f then observing $f(D_0)$ for the initial set of demand shifters D_0 selected by the initialization. For each following iteration, the posterior probability distribution on $f(D)$ is updated as a function of all previously evaluated values in D and a surrogate function is used to model the objective. An acquisition function is used to identify the next D_i that minimizes the lower confidence bound, negative expected improvement, or negative probability of improvement over the posterior distribution, representing the next D_i that is likely to reduce both the value of f and the uncertainty in the surrogate function.[31] The Bayesian optimization model is implemented using the scikit-optimize Optimizer class in Python,[32] with Latin hypercube initialization and a gradient boosted regression trees surrogate model, with the acquisition parameters κ and ξ set to 1 to balance exploration with exploitation. This implementation uses default or near-default values from the scikit-optimize package, with defaults updated only when the initial implementation performed poorly. No hyperparameter optimization was performed due to the relative simplicity of the optimization problem. Because the optimization was performed within each year individually using separate models and could not be performed on forward-looking data, model overfitting was not considered problematic. Demand shifter values for each commodity and year ($\alpha_{y,c}$) are permitted to vary between $\alpha_{\min,c,y}^{0.8}$ and $\alpha_{\max,c,y}^{1.2}$, with $\alpha_{\min,c,y}$ and $\alpha_{\max,c,y}$ being the minimum and maximum values of the demand shifter corresponding to the demand curve intersecting the lowest-price and highest-price points on the supply surface respectively for commodity c in year y.

The demand constant values were tuned for each year and commodity to minimize the score value given by Equation 6 in the main text, varying the value of the price weight variable because the minimization of both production and price error simultaneously produces a multi-objective optimization. Varying the price weight value allows us to establish the Pareto front, or the set of Pareto efficient solutions, defined as all points where any improvement in one variable corresponds with poorer performance for another variable. If we simply compute the mean absolute percentage error over the historical tuning period for production and price across all three commodities and sum over the commodities, we can find the price-production Pareto front, where any decrease in the MAPE for production coincides with an increase in MAPE for price. The results of this exercise are shown in Supplementary Figure 9 for the 2D and 4D approaches.

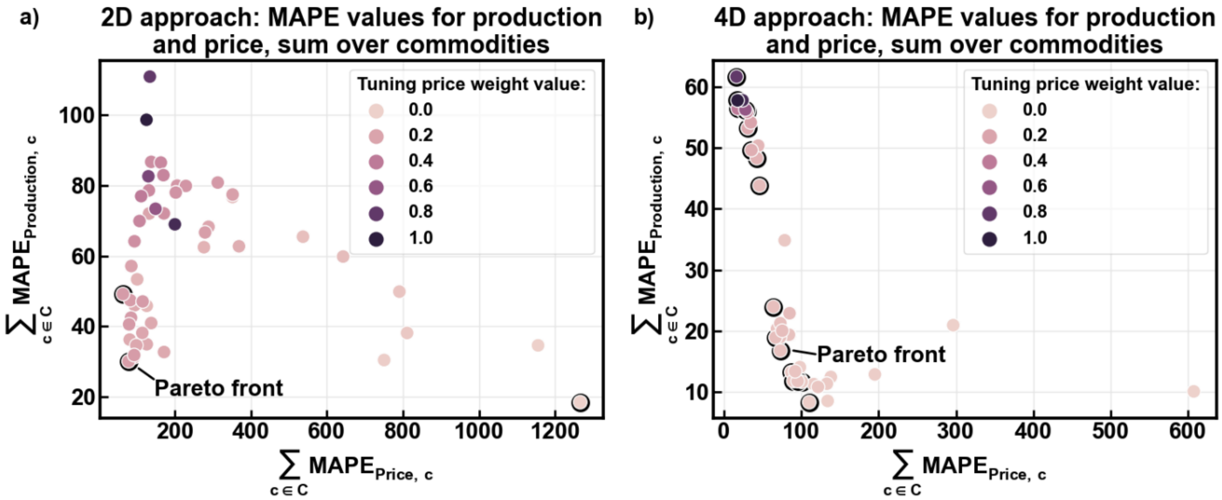

Supplementary Figure 9. Mean absolute percentage error (MAPE) values for each tuning run, summed over all commodities, and plotted for production MAPE on the y-axis and price MAPE on the x axis, for a) the 2D approach and b) the 4D approach. Points with black outlining are on the Pareto front.

The most optimal solutions across both production and price exist at the “elbow” of the Pareto front, but selection of the final price weight value remains subjective. For 2D, the price weight value of 0.2325 was selected, being the Pareto front scenario with the lowest summed production MAPE (30%) having a summed price MAPE below 100% (79%). For 4D, the price weight value of 0.086 was selected, being the scenario with the lowest summed production MAPE (19%) having s summed price MAPE below 70% (67%).

<u>2 Results</u>

2.1 Performance comparison with other methods

Supplementary Table 2. The lowest-error values for each parameter-commodity combination are bolded.

		Co	Cu	Ni
This study, 2D	Production	12.3%	9.2%	8.6%
	Price	31.0%	32.7%	**15.1%**
This study, 4D	Production	7.2%	**6.1%**	**5.6%**
	Price	**21.3%**	**22.1%**	23.5%
Nguyen et al. (2021)	Production	**5.1%**	7.2%	7.1%
	Price	48.8%	27.9%	19.1%

Supplementary References

1. S&P Global Market Intelligence. S&P Capital IQ Pro Metals & Mining Data, Subscription. https://www.capitaliq.spglobal.com/ (2023).
2. Nguyen, R. T., Eggert, R. G., Severson, M. H. & Anderson, C. G. Global Electrification of Vehicles and Intertwined Material Supply Chains of Cobalt, Copper and Nickel. *Resour Conserv Recycl* **167**, (2021).
3. Cobalt Institute. *Cobalt Market Report 2021*. https://www.cobaltinstitute.org/wp-content/uploads/2022/05/FINAL_Cobalt-Market-Report-2021_Cobalt-Institute-1.pdf (2022).
4. Darton Commodities Ltd. *Cobalt Market Review 2020-2021*. https://darton.marex.com/ (2021).
5. Proxima Platform. Cobalt Interactive Data 2023 Q4. *Project Blue* https://proxima.projectblue.com/#/ (2023).
6. Roskill Information Services Ltd. *Cobalt: Outlook to 2029, 15th Edition*. Note: Roskill Information Services Ltd. was acquired by WoodMackenzie in 2021 and legacy reports may be available upon request. https://www.woodmac.com/industry/metals-and-mining/cobalt-product-suite/ (2019).
7. Sun, X., Hao, H., Liu, Z., Zhao, F. & Song, J. Tracing global cobalt flow: 1995–2015. *Resour Conserv Recycl* **149**, 45–55 (2019).
8. International Copper Study Group. *The World Copper Factbook 2018*. https://icsg.org/publications-list/ (2018).
9. International Copper Study Group. *The World Copper Factbook 2021*. https://icsg.org/publications-list/ (2021).
10. International Copper Study Group. *The World Copper Factbook 2012*. https://icsg.org/publications-list/ (2012).
11. International Copper Study Group. *ICSG Statistical Yearbook 2018*. https://icsg.org/publications-list/ (2018).
12. Klose, S. & Pauliuk, S. Sector-level estimates for global future copper demand and the potential for resource efficiency. *Resour Conserv Recycl* **193**, 106941 (2023).
13. Elshkaki, A., Reck, B. K. & Graedel, T. E. Anthropogenic nickel supply, demand, and associated energy and water use. *Resour Conserv Recycl* **125**, 300–307 (2017).
14. International Nickel Study Group. *World Nickel Factbook 2021*. (2021).
15. Proxima Platform. Nickel Interactive Data 2023 Q4. *Project Blue* https://proxima.projectblue.com/#/ (2023).
16. S&P Global Market Intelligence. S&P Capital IQ Pro Capital Costs Data, Subscription. https://www.capitaliq.spglobal.com/ (2023).
17. Gulley, A. L. China, the Democratic Republic of the Congo, and artisanal cobalt mining from 2000 through 2020. *Proc Natl Acad Sci U S A* **120**, e2212037120 (2023).
18. WoodMackenzie. 2021 Cobalt Outlook. *WoodMackenzie* https://my.woodmac.com/ (2022).
19. U.S. Geological Survey. *Nickel Statistics [through 2018; Last Modified May 3, 2021] in Kelly, T.D., and Matos, G.R., Comps, Historical Statistics for Mineral and Material Commodities in the United States (2023 Version): U.S. Geological Survey Data Series 140, Accessed September 2023 at Https://Www.Usgs.Gov/Centers/National-Minerals-Information-Center/Historical-Statistics-Mineral-and-Material-Commodities*. (2021).
20. U.S. Geological Survey. *Copper Statistics [through 2018; Last Modified May 3, 2021] in Kelly, T.D., and Matos, G.R., Comps, Historical Statistics for Mineral and Material Commodities in the United*

States (2023 Version): U.S. Geological Survey Data Series 140, Accessed September 2023 at Https://Www.Usgs.Gov/Centers/National-Minerals-Information-Center/Historical-Statistics-Mineral-and-Material-Commodities. (2021).

21. U.S. Geological Survey. *Cobalt Statistics [through 2019; Last Modified March 31, 2021] in Kelly, T.D., and Matos, G.R., Comps, Historical Statistics for Mineral and Material Commodities in the United States (2023 Version): U.S. Geological Survey Data Series 140, Accessed September 2023 at Https://Www.Usgs.Gov/Centers/National-Minerals-Information-Center/Historical-Statistics-Mineral-and-Material-Commodities*. (2021).
22. U.S. Geological Survey. Mineral Commodity Summaries 2021. *U.S. Geological Survey* https://pubs.usgs.gov/publication/mcs2021 (2021).
23. U.S. Geological Survey. Mineral Commodity Summaries 2022. *U.S. Geological Survey* https://pubs.usgs.gov/publication/mcs2022 (2022).
24. Pedregosa, F. *et al.* Scikit-learn: Machine Learning in Python. *Journal of Machine Learning Research* **12**, 2825–2830 (2011).
25. Cohen, A. fuzzywuzzy. *PyPI - Python Package Index* (2020).
26. Eng, D. P.-Int. J. Adv. Sci. Res. Similarity based information retrieval using Levenshtein distance algorithm. *International Journal of Advances in Scientific Research and Engineering* **6**, (2020).
27. Ha, J., Kose, M. A. & Ohnsorge, F. *One-Stop Source A Global Database of Inflation*. http://www.worldbank.org/prwp. (2021).
28. Ryter, J., Fu, X., Bhuwalka, K., Roth, R. & Olivetti, E. Assessing recycling, displacement, and environmental impacts using an economics-informed material system model. *J Ind Ecol* **26**, 1010–1024 (2022).
29. Hunter, J. D. Matplotlib: A 2D graphics environment. *Comput Sci Eng* **9**, 90–95 (2007).
30. Shojaeddini, E., Alonso, E. & Nassar, N. T. Estimating price elasticity of demand for mineral commodities used in Lithium-ion batteries in the face of surging demand. *Resour Conserv Recycl* **207**, 107664 (2024).
31. Frazier, P. I. A Tutorial on Bayesian Optimization. *arXiv* https://doi.org/10.48550/arXiv.1807.02811 (2018).
32. Head, T. scikit-optimize · PyPI. *PyPI* https://pypi.org/project/scikit-optimize/ (2021).
